# Genetic variation in *CCR2* and *CXCL12* genes impacts on CD4 restoration in patients initiating cART with advanced immunesupression

**DOI:** 10.1371/journal.pone.0214421

**Published:** 2019-03-28

**Authors:** Clara Restrepo, Mónica Gutierrez-Rivas, Yolanda M. Pacheco, Marcial García, Julià Blanco, Luz M. Medrano, María A. Navarrete-Muñoz, Félix Gutiérrez, Pilar Miralles, David Dalmau, Juan Luis Gómez, Miguel Górgolas, Alfonso Cabello, Salvador Resino, José M. Benito, Norma Rallón, on behalf of CoRIS and the HIV Biobank integrated in the Spanish AIDS Research Network Project RIS/EPICLIN 10_2015

**Affiliations:** 1 HIV and Viral Hepatitis Research Laboratory, Instituto de Investigación Sanitaria-Fundación Jiménez Díaz, Universidad Autónoma de Madrid (IIS-FJD, UAM), Madrid, Spain; 2 Hospital Universitario Rey Juan Carlos, Móstoles (Madrid), Spain; 3 Instituto de Salud Carlos III, Madrid, Spain; 4 Laboratory of Immunology, Instituto de Biomedicina de Sevilla (IBiS)/UGC Clinical Laboratories, Hospital Universitario Virgen del Rocío, Sevilla, Spain; 5 IrsiCaixa AIDS Research Institute, Badalona, Spain; 6 Hospital General Universitario de Elche & University Miguel Hernández, Alicante, Spain; 7 Hospital General Universitario Gregorio Marañón, Madrid, Spain; 8 Hospital Universitari Mutua Terrasa, Terrasa, Spain; 9 Hospital Universitario de Canarias, Santa Cruz de Tenerife, Spain; 10 Hospital Universitario Fundación Jiménez Díaz, Madrid, Spain; Western Sydney University, AUSTRALIA

## Abstract

**Objective:**

We investigated the association of genetic polymorphisms in chemokine and chemokine receptor genes with poor immunological recovery in HIV patients starting combined antiretroviral therapy (cART) with low CD4 T-cell counts.

**Methods:**

A case-control study was conducted in 412 HIV-infected patients starting cART with CD4 T-cell count <200 cells/μL and successful viral control for two years. CD4 count increase below 200 cells/μL after two years on cART was used to define INR (immunological non-responder) patients. Polymorphisms in *CXCL12*, *CCL5* and *CCR2* genes were genotyped using sequenom’s MassARRAY platform.

**Results:**

Thirty two percent (134/412) of patients were classified as INR. After adjusting by age, route of HIV infection, length of infection before cART and viral hepatitis coinfection, *CCR2* rs1799864-AG genotype was significantly associated with INR status (OR [95% CI]: 1.80 [1.04–3.11]; p = 0.04), and *CXCL12* rs1801157-TT genotype showed a trend (OR [95% CI]: 2.47 [0.96–6.35]; p = 0.06).

**Conclusions:**

*CCR2* rs1799864-AG or *CXCL12* rs1801157-TT genotypes influence on the probability of poor CD4 recovery in the population of HIV patients starting cART with low CD4 counts. Genotyping of these polymorphisms could be used to estimate the risk of poor CD4 restoration, mainly in patients who are diagnosed late in the course of infection.

## Introduction

The progressive loss of CD4 T cells is the hallmark of HIV pathogenesis. Treatment of HIV infection with combination antiretroviral therapy (cART) usually results in suppression of viral replication to undetectable levels and increasing CD4 T cell counts. However, despite suppression of viral replication, approximately 30% of HIV-infected patients do not achieve an optimal CD4 T-cell recovery [1,2] and these individuals are referred to as immunological non-responders (INR) [3]. This phenomenon is of clinical relevance because the persistently low CD4 T cell counts are associated with a high risk of disease progression, AIDS and non-AIDS clinical events and death [4]. Mechanisms underlying the INR status are not well understood. Nadir CD4 T cell count (defined as the lowest point to which the CD4 count has dropped) at the beginning of cART is the most common determinant of poor immune recovery [5,6] representing an important concern because of the high proportion of patients showing CD4 T-cell counts below 350 cells/mm3 or even <200 cells/mm3 at the moment of diagnosis [7].

Several other factors have been involved on poor CD4 recovery upon successful cART such as older age [8], co-infection with HCV [9], high CD4+ T cell apoptosis [6], chronic immune activation [10,11], or poor thymic output [12]. Moreover, some studies have suggested that variability between individuals in the capacity of the immune system to recover after antiretroviral therapy is attributed to the genetic variations among HIV-infected patients [13]. Polymorphisms in genes encoding different immune-regulating molecules [14], and molecules involved in T-cell homeostasis [15] and cellular metabolism [16] have been associated with the extent of CD4 T cell recovery during cART.

Further, genetic polymorphisms in genes coding for chemokines and chemokine receptors have been demonstrated to influence both HIV transmission and disease progression [17–19]. Genetic polymorphisms in chemokines CCL5 (RANTES) and CXCL12 (SDF1), which act as potent blockers of HIV infection by competing with the virus for binding to *CCR5* and *CXCR4* receptors respectively, have been described to be associated with HIV disease progression. For instance, C allele of the intronic polymorphism rs2280789 in the *CCL5* gene has been associated with accelerated progression to AIDS by down regulating *CCL5* gene transcription [20]. Homozygosity for A allele of polymorphism rs1801157 in *CXCL12* gene has been associated with slower progression to AIDS [21], although this finding has not been confirmed by others [18, 22]. Regarding genetic variants in chemokine receptors, heterozygosity or homozytosity of A allele of polymorphism rs1799864 (V64I) in the *CCR2* gene has been associated with slower progression to AIDS [23].

The contribution of the genetic variants mentioned above in the context of antiretroviral treatment has been less explored and the results are unclear [24–26]. Furthermore, the potential influence of these genetic variants in the phenomenon of immunological non response in the special population of patients starting cART with low CD4 counts has not been explored so far. Our goal in this study was to investigate the association of host genetic polymorphisms in *CCL5*, *CXCL12* and *CCR2* genes with the phenomenon of immunological discordance in a cohort of HIV infected patients starting cART with very low CD4 T-cell counts and maintaining complete suppression of viral replication after treatment.

## Materials and methods

### Study population

**Fig 1:**
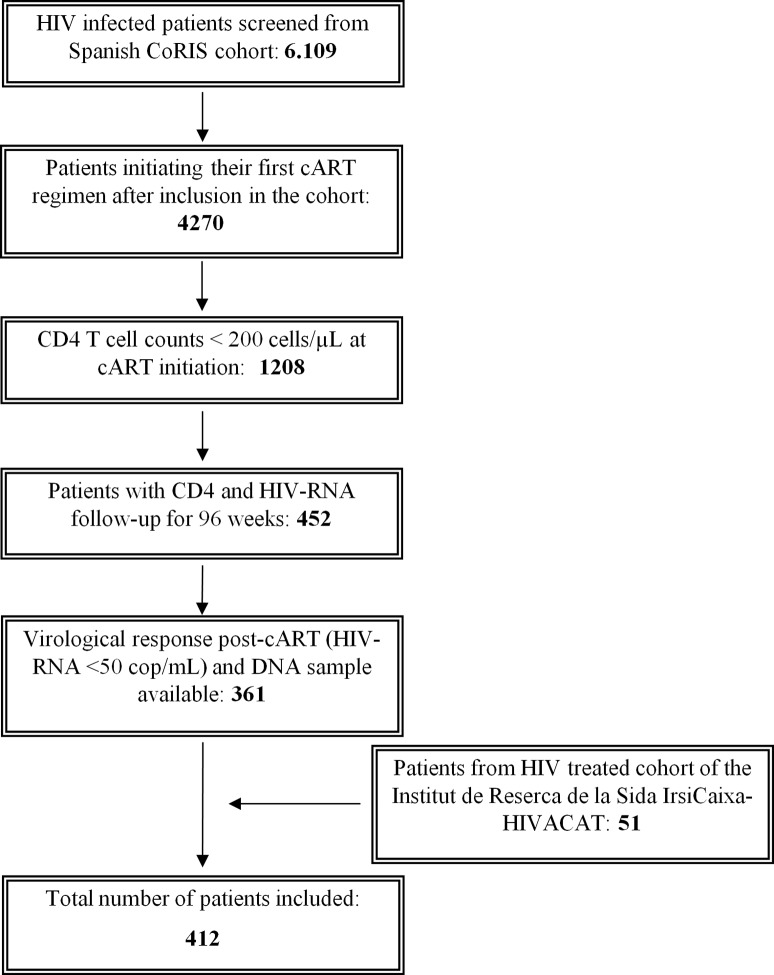
Flow chart of inclusion criteria of patients. Flow chart showing the inclusion criteria and the sequential strategy of selection of patients included in the study. Numbers inside the boxes indicate the number of patients selected after each step in the selection process.

This is a retrospective, case-control study for which we screened 6109 HIV infected patients included in the cohort of the Spanish AIDS Research Network (CoRIS), a multicentre cohort with clinical data and biological samples of HIV-positive subjects launched in 2004 [27,28]. The majority of samples were kindly provided by the Spanish HIV BioBank integrated in the Spanish AIDS Research Network (RIS) [29]. The inclusion criteria were (Fig 1): a) patients initiating their first cART regimen; *b)* CD4 T-cell counts at the initiation of cART <200 cells/μL; *c)* complete viral suppression (plasma HIV-RNA <50 copies/mL) during 2 years of therapy; *d)* regular follow up of CD4 counts and HIV viral load; e) having a DNA sample available for analysis. Using these inclusion criteria, 361 patients were selected from the CoRIS cohort. In addition, a cohort of 51 patients meeting the same inclusion criteria was provided by the HIV cohort of the Institut de Recerca de la Sida IrsiCaixa-HIVACAT, Institut d’Investigació en Ciènces de la Salut Germans Trias i Pujol (Badalona, Spain). Protocols were approved by institutional ethical committees (Germans Trias I Pujol Hospital ethical committee and CoRIS ethical committee) and all individuals provided written an informed consent to participate in the study.

### Polymorphisms genotyping

We selected three chemokine and chemokine receptor polymorphisms: rs2280789 located in *CCL5* gene, rs1801157 located in *CXCL12* gene and rs1799864 located in *CCR2* gene. All of these polymorphisms have a minor allele frequency (MAF) higher than 5% in the CEU (Utah residents with ancestry from Northern and Western Europe) and Tuscan (Italy) populations according to 1000 Genomes Project database (http://www.internationalgenome.org/) [30]. Moreover, as a reference group, a control healthy population consisting of 107 individuals of Iberian origin (Iberian populations in Spain, IBS) was obtained from this same database.

Genomic DNA was extracted from cryopreserved peripheral blood mononuclear cells (PBMCs) using a QIAamp DNA kit (Qiagen, Barcelona, Spain) following manufacturer’s instructions. DNA samples were genotyped at the Spanish National Genotyping Center (CeGen; http://www.usc.es/cegen/). Genotyping was performed by using sequenom’s MassARRAY platform (San Diego, CA, USA) using the Iplex Gold assay design system. The quality control was performed according to the CeGen criteria and all samples were made in duplicate on each plate. Moreover, a negative control to exclude DNA contamination and positive control to ensure a technically correct laboratory process were included in each assay plate.

### Outcome variable

CD4 T-cell count increase (ΔCD4) below or above 200 cells/μL after two years on cART and suppressed viral replication was the outcome variable. Immunological non-responders (INR) patients were defined as patients showing a ΔCD4 <200 cells/μL, and immunological responders (IR) patients as patients showing a ΔCD4 ≥200 cells/μL.

### Statistical analysis

To describe study population, median and interquartile range (IQR) was employed. Nonparametric tests (Mann-Whitney U Test for continuous variable and Chi-square (x^2^) or Fisher`s exact test for categorical variables) were used to compare different groups of subjects. Allelic and genotypic frequencies and association studies for individual polymorphisms were performed using the web tool SNPStats (https://www.snpstats.net/start.htm) [31]. SNPs were evaluated for Hardy-Weinberg equilibrium (HWE), considering p >0.05. Association of the different SNPs variants with INR status was tested using five different models: recessive, dominant, over-dominant, log-additive and co-dominant. Multivariate logistic regression analysis was used to test the association of *CCL5*, *CXCL12* and *CCR2* SNPs with INR status, adjusting by other clinical and epidemiological variables that showed significant association with INR status. Statistical analyses were performed using SPSS software version 15 (SPSS Inc., Chicago, IL, USA). All p-values were considered significant when <0.05.

## Results

### Study population

A total of 412 patients meeting all the inclusion criteria were analyzed. Of them, 134 patients were classified as INR because they had a CD4 increase (ΔCD4) below 200 cells/μL after 2 years on cART. The remaining 278 patients were classified as IR because they showed a ΔCD4 equal or above 200 cells/μL after the same period of cART. As expected, there were significant differences in ΔCD4 between INR and IR groups of patients (133 [73–174] and 342 [267–467] cells/μL in INR and IR groups respectively; p<0.0001). Table 1 shows different characteristics in the total population of patients and in INR and IR groups. There were no differences in CD4 T-cell count pre-cART between INR and IR (99 [41–148] and 107 [41–162] cells/μL respectively, p = 0.4). However, significant differences between INR and IR groups were observed for age (42 [36–48] vs. 39 [32–47] years respectively, p = 0.009); length of infection before cART (1 [1–2] and 1 [1–1] years respectively, p = 0.029); prevalence of infection with hepatitis viruses (18% vs. 10% respectively, p = 0.018); and transmission category with more prevalence of injected drug users in INR than IR (22% vs. 11% respectively, p = 0.003).

**Table 1:** Characteristics of study population.

Characteristics	All patients (n = 412)	INR patients (n = 134)	IR patients (n = 278)	p-values*
Age (years)	40 [34–47]	42 [36–48]	39 [32–47]	**0.009**
Length of HIV infection before cART (months)	3 [1–23]	3 [1–56]	3 [1–14]	**0.045**
CD4 counts at baseline (cells/μL)	104 [41–159]	99 [41–148]	107 [41–162]	0.405
CD4 increase after 2 years on cART (cells/μL)	268 [177–400]	133 [73–174]	342 [267–467]	**<0.0001**
Coinfection with HCV or HBV. n (%)	52/412 (13)	24/134 (18)	28/278 (10)	**0.018**
Transmission category				**0.003**
Sexual	329/385 (85)	93/120 (78)	236/265 (89)	
Parenteral	56/385 (15)	27/120 (22)	29/265 (11)	
Gender (male). N° (%)	324/412 (79)	112/134 (84)	212/278 (76)	0.089
cART regimen. N° (%)				0.721
PI-based regimen	127/411 (31)	40/134 (30)	87/277 (31)	
Non PI-based regimen	284/411 (69)	94/134 (70)	190/277 (69)	
Ethnic origin. N° (%)				0.062
Caucasian	318/394 (81)	108/124 (87)	210/270 (78)	
African	25/394 (6)	7/124 (6)	18/270 (7)	
Latin American	51/394 (13)	9/124 (7)	42/270 (15)	

Data are given as median and interquartile range [IQR], unless otherwise indicated. **INR**: immunological non responders. **IR**: immunological responders. **cART**: combination antiretroviral therapy. **HCV**: hepatitis C virus. **HBV**: hepatitis B virus.

* **p-values** for the comparison between IR and INR patients (Mann-Whitney U test or Chi-square test as appropriate)

### Alleles and genotypes distribution of *CCL5* rs2280789, *CXCL12* rs1801157, and *CCR2* rs1799864 polymorphisms

The distribution of *CCL5* rs2280789, *CCR2* rs1799864, and *CXCL12* rs1801157 alleles and genotypes in INR and IR patients is shown in Table 2. All three polymorphisms analyzed had a minimum allele frequency (MAF)> 0.05 and satisfied the HWE (p >0.05). Overall, both alleles and genotypes distribution of all three polymorphisms was similar between groups.

**Table 2:** Summary of alleles and genotype frequencies for CCL5 rs2280789, CCR2 rs1799864 and CXCL12 rs1801157 polymorphims in the study population.

SNP alleles and genotypes	All patients (n = 412)	IR (n = 278)	INR (n = 134)	p-value
**CCL5 rs2280789**				
**Alleles**				
A	86%	86%	85%	0.30
G	14%	14%	15%	
**Genotypes**				
AA	75%	76%	72%	0.54
AG	23%	21%	26%	
GG	2%	3%	2%	
HWE (p-value)	0.41	0.13	0.74	
**CXCL12 rs1801157**				
**Alleles**				
C	78%	79%	76%	0.20
T	22%	21%	24%	
**Genotypes**				
CC	61%	62%	60%	0.12
CT	34%	35%	32%	
TT	5%	3%	8%	
HWE (p-value)	1	0.36	0.23	
**CCR2 rs1799864**				
**Alleles**				
G	90%	91%	89%	0.20
A	10%	9%	11%	
**Genotypes**				
AA	1%	1%	0%	0.09
GA	18%	16%	22%	
GG	81%	83%	78%	
HWE (p-value)	0.78	0.47	0.37	

**INR:** immunological non responders patients; **IR:** immunological responders patients; **HWE**: Hardy-Weinberg equilibrium.

p-values for the comparison between IR and INR patients (Chi-square test)

The *CCL5* rs2280789 genotype GG was very rare in both IR (3%) and INR (2%) patients. Seventy six percent of IR and 72% of INR where homozygous for rs2280789 A allele, whereas 21% percent of IR and 26% of INR patients were heterozygous for rs2280789 (p = 0.54). Regarding *CXCL12* rs1801157, genotype TT frequency was low in both IR (3%) and INR (8%) patients. Sixty two percent of IR and 60% of INR patients carried rs1801157 CC genotype, whereas 35% of IR and 32% of INR patients carried rs1801157 genotype CT (p = 0.12). The distribution of *CCR2* rs1799864 genotypes was: 1% of IR and none of INR patients carried rs1799864 AA genotype. Eighty three percent of IR and 78% of INR patients were homozygous for rs1799864 G allele, whereas the proportion of individuals carrying rs1799864 AG genotype was slightly higher in INR compared to IR patients (22% and 16% respectively, p = 0.09).

In addition, alleles and genotypes frequencies of these three polymorphisms in our study population were very similar to those reported by 1000 genomes database in the Iberian populations in Spain (IBS) [30], except for the *CCL5* rs2280789 AA genotype (p = 0.04) (S1 Table).

### Association of *CCL5* rs2280789, *CXCL12* rs1801157 and *CCR2* rs1799864 polymorphisms with INR status

Using SNPstats web tool [31], we first tested the best association model with INR status for the different SNPs analyzed. The best models of association fitting our data were a recessive model for *CXCL12* rs1801157 and an over-dominant model for *CCR2* rs1799864 polymorphisms, whereas *CCL5* rs2280789 showed no association with INR with any of the models tested. According to these models of association, patients were stratified into two groups according to *CXCL12* rs1801157 polymorphism into those carrying TT genotype and those carrying CT or CC genotype; and according to *CCR2* rs1799864 polymorphism into those carrying AG genotype and those carrying AA or GG genotypes.

Next, we performed both univariate and multivariate logistic regression analysis. By univariate logistic regression, *CXCL12* rs1801157 TT genotype (OR: 2.40 [0.96–6.13]; p = 0.05) and *CCR2* rs1799864 AG genotype (OR: 1.50 [0.91–2.58]; p = 0.10) showed a trend to be associated to INR status (Table 3). However, after correcting by age, length of infection, co-infection with hepatitis viruses and transmission route, variables that presented significant differences between INR and IR groups (Table 1), the multivariate logistic analysis showed that *CCR2* rs1799864 AG genotype was significantly and independently associated to INR status (OR: 1.80 [1.04–3.11]; p = 0.036) and *CXCL12* rs1801157 TT genotype showed a clear trend (OR: 2.47 [0.96–6.13]; p = 0.063) (Table 3).

**Table 3:** Association of CXCL12 rs1801157 (recessive inheritance model) and CCR2 rs1799864 (over-dominant inheritance model) genotypes with INR status.

	Frequencies			Univariate analysis				Multivariate analysis		
Genotypes	IR (n = 278)	INR (n = 134)		OR [95% CI]		p-value		OR [95% CI]		p-value^a^
**CXCL12 rs1801157**										
TT	3% (9/278)	8% (10/134)		2.4 [0.96–6.13]		0.05		2.47 [0.96–6.35]		0.063
CC/CT	97% (269/278)	92% (123/134)		1				1		
**CCR2 rs1799864**										
AG	16% (44/278)	22% (30/134)		1.5 [0.91–2.58]		0.10		1.80 [1.04–3.11]		0.036
GG/AA	84% (234/278)	78% (104/134)		1				1		

**IR:** immunological responders patients, **INR:** immunological non-responders patients, **OR:** Odds Ratio; **CI:** confidential interval

^a^ p-values were calculated by logistic regression adjusted by age, length of infection before cART, transmission route, and hepatitis co-infection.

## Discussion

The immunological non-responders are a group of HIV patients who do not achieve an optimal CD4 T-cell recovery despite receiving cART and maintaining complete viral suppression [3]. Understanding the host factors influencing this impaired immune recovery despite cART, may be critical to design appropriate strategies aimed to improve the immune status in these patients.

Given the pivotal role of chemokines and chemokine receptors in the lymphocyte traffic and its known effect in HIV disease progression, in this study we explored the potential association of *CCL5* rs2280789, *CXCL12* rs1801157, and *CCR2* rs1799864 polymorphisms with CD4 T-cell recovery in a particular cohort of 412 HIV infected individuals who began cART with low CD4 T-cell counts and who maintained complete suppression of HIV plasma viremia during 2 years on treatment. Interestingly, our results showed that *CCR2* rs1799864 AG and *CXCL12* rs1801157 TT genotypes were associated with a higher probability of having poor CD4 T-cell recovery in HIV patients despite complete viral suppression with cART. No significant association was found for *CCL5* rs2280789 polymorphism, suggesting that this polymorphism has no effect on CD4 T-cell recovery after cART, in contrast with the results of a previous study that found a significant association for this same polymorphism with HIV disease progression in the absence of therapy [20]. However, given the different scenarios (CD4 T-cell recovery after therapy versus CD4 T-cell loss in the absence of therapy), the results of our study are not contradictory with the previous study.

The role of *CCR2* gene polymorphisms on CD4 T-cell recovery in the context of cART has been poorly studied and the results still remain unclear. The majority of studies investigating the effect of the *CCR2* gene polymorphisms in progression of HIV patients receiving antiretroviral therapy have reported no influence of rs1799864 genotypes on CD4 T-cell gains [25, 26, 32, 33]. However, another study reported that carriage of CCR2 rs1799864-A allele was associated with immunological outcome after initiation of cART [34]. In partial agreement with this last study, results of our study showed a clear association between *CCR2* rs1799864 genotype and the probability of presenting an INR phenotype after cART. However, the association we found was with *CCR2* rs1799864 AG genotype and not with *CCR2* rs1799864 A allele, probably due to the very low prevalence of *CCR2* rs1799864 AA genotype in our study population (only 1% of the IR patients and none in the INR patients). Moreover, Rigato et al found that A allele was associated with better inmmunological outcome, in contrast with our results showing a poorer outcome in patients with AG genotype. Interestingly, Rigato *et al*.[34] also found lower level of CD4 restoration in those individuals carrying *CCR2* rs1799864 AG genotype, although differences did not reach significance likely due to a much more limited sample size of their study compared to ours. Surprisingly, the protective effect of *CCR2* rs1799864 A allele reported by Rigato et al was revealed only when combined with CCR5-D32 polymorphism [34], what is in contrast with a poorer outcome of AG individuals compared with AA individuals observed in this same study [34]. Clearly the very low prevalence of rs1799864 AA genotype in our study precludes finding the existence of an association between rs1799864 A allele and INR phenotype and thus further studies with larger cohorts of patients, including a greater number of individuals carrying rs1799864 AA genotype, are warranted to analyze the true role of this allele on CD4 restoration after cART.

Regarding the discordant findings between our study and previous studies reporting no association of CD4 restoration with CCR2 gene variation, differences in the size of cohorts, length of follow-up, study design, pre-cART CD4 counts and ethnic background, among others, may be involved in these discordant findings. It is important to highlight that in contrast to previous studies analyzing *CCR2* polymorphisms, our study included a homogeneous population in terms of cART exposure (all patients were naïve for cART when included in the study) and of baseline CD4 counts (including only patients who started treatment with very low CD4 T-cell counts, <200 cells/μL). It should also be taken into account that we performed the association analysis adjusting by clinical and epidemiological variables associated with the phenomenon of INR such as age [8], co-infection with HCV [9] or length of infection before treatment, what gives a strong significance to our findings.

The rs1799864 variation results in amino acid substitution from Valine to Isoleucine (V64I) at position 64 in the transmembrane domain of *CCR2* protein [23]. There is not a consensus about the true role of *CCR2* polymorphisms in the HIV disease progression. Some HIV virus strains can use this chemokine receptor to infect cells and it is unclear why a single amino acid substitution in a minor co-receptor could affect HIV disease progression. Curiously, an in vitro study has shown that both wild-type *CCR2* and the mutated (V64I) version of the receptor were equally permissive for HIV infection regardless of viral tropism, were expressed at similar levels on the cell surface and were equally effective acting as viral co-receptors and as chemokine receptors, suggesting that this variation probably has neither a protective nor a deleterious effect in the course of HIV infection [35]. A potential explanation could be that this polymorphism acts simply as a tag-SNP that is in linkage disequilibrium with another polymorphism responsible for this association. In line with this, a previous study reported that rs1799864 *CCR2* polymorphism is in complete linkage disequilibrium with a C to T substitution on CCR5 regulatory region at position 59653 [36]. All individuals carrying the A allele at rs1799864 *CCR2* SNP also carried the T allele at *CCR5* promoter polymorphism [36]. Regarding how the C to T substitution relates to CCR5 regulation, no conclusive results have been reported so far, although Kostrikis et al in preliminary *in vitro* experiments showed no effect of CCR2 rs1799864 SNP on the levels of CCR5 expression [36].

Regarding *CXCL12* rs1801157 polymorphism, also evaluated in our study, the multivariate analysis revealed that the TT genotype had a trend toward being associated with INR status in our cohort of HIV treated patients. The lack of statistical significance is likely due to the very low prevalence of TT genotype and thus increasing the number of individuals with this rare genotype could potentially lead to significance for this association, although this would require a much larger cohort of patients. In line with our results, some reports have suggested that this genotype is associated with accelerated HIV disease progression in untreated patients [22, 37]. Daar *et al*. showed that untreated HIV infected individuals carrying *CXCL12* rs1801157 T allele showed a faster CD4 decline and an increased likelihood of harboring CXCR4 tropic viruses which has been associated with an accelerated disease progression [37]. Previous studies addressing the association of *CXCL12* rs1801157 variation with CD4 restoration after cART have yielded discordant results, with some of them reporting a significant association [26, 33] but not others [25, 34]. Several differences between our study and the above mentioned studies preclude a fair comparison. Differences in the study design such baseline CD4 T-cell counts before initiating cART, criteria to evaluate CD4 restoration, length of follow up and small size of cohorts evaluated may explain the seemingly contradictory results. Since we used a large cohort of patients homogeneous in terms of pre-cART CD4 counts and were able to perform a multivariate analysis adjusting for several covariables that could impact on the CD4 restoration, we feel confident with the conclusions of our study.

The CXCL12 is a proinflamatory cytokine having pleiotropic effects on chemotaxis, angiogenesis, immune response and tumor metastasis. CXCL12 is the natural ligand of CXCR4, the co-receptor of X4 tropic HIV infection, thus CXCL12 can inhibit entry of HIV into the CD4 cells [38]. Regarding the biological mechanisms underlying the impact of *CXCL12* rs1801157 T allele on the HIV disease progression [22, 37], a previous study reported lower CXCL12 mRNA expression in carriers of T allele [39]. In line with this, another study found a clear association between homozygosity for T allele and low plasma levels of CXCL12 protein that could diminish the competition with HIV to CXCR4 binding [40]. Interestingly, it has also been reported that patients with *CXCL12* rs1801157 T allele are more likely to carry X4-tropic viruses [37]. Findings of these studies could explain the association of CXCL12 rs1801157 TT genotype with poor CD4 T-cell recovery in HIV patients on cART that we found in our study.

In summary, the data of our study shows that in HIV patients starting cART with low CD4 counts, *CCR2* rs1799864 AG and/or *CXCL12* rs1801157 TT genotypes influences the probability of poor CD4 T-cells recovery in spite of successful suppression of HIV replication. Interestingly, this impact is independent of other well known factors associated to the INR status and thus genotyping of these two polymorphisms could add prognostic value to detect those patients with higher risk of poor CD4 restoration, in a population of patients that is growing due to the increasing proportion of late HIV diagnosis.

## Supporting information

S1 Text Centers and investigators participating in CoRIS. (PDF)

S1 Table Alleles and genotypes frequencies for *CCL5* rs2280789, *CXCR2* rs1799864 and *CXCL12* rs1801157 polymorphisms in the study population compared with Iberian Populations (IBS) in Spain from 1000 genomes database (http://www.internationalgenome.org/). (PDF)

## References

[1] Gazzola L, Tincati C, Bellistrì GM, Monforte AD, Marchetti G. The absence of CD4+ T cell count recovery despite receipt of virologically suppressive highly active antiretroviral therapy: clinical risk, immunological gaps, and therapeutic options. Clin Infect Dis. 2009; 48(3):328–337. 10.1086/595851 19123868

[2] Kelley CF, Kitchen CM, Hunt PW, Rodriguez B, Hecht FM, Kitahata M, et al Incomplete peripheral CD4+ cell count restoration in HIV-infected patients receiving long-term antiretroviral treatment. Clin Infect Dis. 2009;48(6):787–794. 10.1086/597093 19193107 PMC2720023

[3] Gaardbo JC, Hartling HJ, Gerstoft J, Nielsen SD. Incomplete immune recovery in HIV infection: mechanisms, relevance for clinical care, and possible solutions. Clin Dev Immunol. 2012; 2012:670957 10.1155/2012/670957 22474480 PMC3312328

[4] Zoufaly A, Cozzi-Lepri A, Reekie J, Kirk O, Lundgren J, Reiss P, et al Immuno-virological discordance and the risk of non-AIDS and AIDS events in a large observational cohort of HIV-patients in Europe. PLoS One. 2014;9(1):e87160 10.1371/journal.pone.0087160 24498036 PMC3909048

[5] Moore RD, Keruly JC. CD4+ cell count 6 years after commencement of highly active antiretroviral therapy in persons with sustained virologic suppression. Clin Infect Dis. 2007;44(3):441–446. 10.1086/510746 17205456

[6] Negredo E, Massanella M, Puig J, Pérez-Alvarez N, Gallego-Escuredo JM, Villarroya J, et al Nadir CD4 T cell count as predictor and high CD4 T cell intrinsic apoptosis as final mechanism of poor CD4 T cell recovery in virologically suppressed HIV-infected patients: clinical implications. Clin Infect Dis. 2010;50(9):1300–1308. 10.1086/651689 20367229

[7] Mocroft A, Lundgren JD, Sabin ML, Monforte Ad, Brockmeyer N, Casabona J, et al Risk factors and outcomes for late presentation for HIV-positive persons in Europe: results from the Collaboration of Observational HIV Epidemiological Research Europe Study (COHERE). PLoS Med. 2013;10(9):e1001510 10.1371/journal.pmed.1001510 24137103 PMC3796947

[8] Appay V, Fastenackels S, Katlama C, Ait-Mohand H, Schneider L, Guihot A, et al Old age and anti-cytomegalovirus immunity are associated with altered T-cell reconstitution in HIV-1-infected patients. AIDS. 2011;25(15):1813–1822. 10.1097/QAD.0b013e32834640e6 21412126

[9] Miller MF, Haley C, Koziel MJ, Rowley CF. Impact of hepatitis C virus on immune restoration in HIV-infected patients who start highly active antiretroviral therapy: a meta-analysis. Clin Infect Dis. 2005;41(5):713–720. 10.1086/432618 16080095

[10] Massanella M, Negredo E, Pérez-Alvarez N, Puig J, Ruiz-Hernández R, Bofill M, et al CD4 T-cell hyperactivation and susceptibility to cell death determine poor CD4 T-cell recovery during suppressive HAART. AIDS. 2010;24(7):959–968. 10.1097/QAD.0b013e328337b957 20177358

[11] Nakanjako D, Ssewanyana I, Mayanja-Kizza H, Kiragga A, Colebunders R, Manabe YC, et al High T-cell immune activation and immune exhaustion among individuals with suboptimal CD4 recovery after 4 years of antiretroviral therapy in an African cohort. BMC Infect Dis. 2011;11:43 10.1186/1471-2334-11-43 21299909 PMC3065409

[12] Li T, Wu N, Dai Y, Qiu Z, Han Y, Xie J, et al Reduced thymic output is a major mechanism of immune reconstitution failure in HIV-infected patients after long-term antiretroviral therapy. Clin Infect Dis. 2011;53(9):944–951. 10.1093/cid/cir552 21960716

[13] Haas DW, Geraghty DE, Andersen J, Mar J, Motsinger AA, D'Aquila RT, et al Immunogenetics of CD4 lymphocyte count recovery during antiretroviral therapy: An AIDS Clinical Trials Group study. J Infect Dis. 2006;194(8):1098–107. 10.1086/507313 16991084

[14] Soria A, Guerini FR, Bandera A, Bolognesi E, Uglietti A, Fusco C, et al KIR-HLA genotypes in HIV-infected patients lacking immunological recovery despite effective antiretroviral therapy. PLoS One. 2011;6(11):e27349 10.1371/journal.pone.0027349 22073315 PMC3207876

[15] Guzmán-Fulgencio M, Berenguer J, Jiménez-Sousa MA, Micheloud D, García-Álvarez M, Bellón JM, et al IL7RA polymorphisms predict the CD4+ recovery in HIV patients on cART. Eur J Clin Invest. 2015; 45(11):1192–1199. 10.1111/eci.12539 26402121

[16] Chan DP, Lee MP, Wong NS, Leung RK, Naftalin CM, Lee SS. Association of immune recovery with hyperlipidaemia and apolipoprotein gene polymorphisms following highly active antiretroviral therapy in a cohort of Chinese HIV patients. BMJ Open. 2016;6(4):e010998 10.1136/bmjopen-2015-010998 27067897 PMC4838726

[17] Arenzana-Seisdedos F, Parmentier M. Genetics of resistance to HIV infection: Role of co-receptors and co-receptor ligands. Semin Immunol. 2006;18(6):387–403. 10.1016/j.smim.2006.07.007 16978874

[18] Ioannidis JP, Rosenberg PS, Goedert JJ, Ashton LJ, Benfield TL, Buchbinder SP, et al Effects of CCR5-Delta32, CCR2-64I, and SDF-1 3'A alleles on HIV-1 disease progression: An international meta-analysis of individual-patient data. Ann Intern Med. 2001;135(9):782–795. 11694103 10.7326/0003-4819-135-9-200111060-00008

[19] Mahajan SD, Agosto-Mojica A, Aalinkeel R, Reynolds JL, Nair BB, Sykes DE, et al Role of chemokine and cytokine polymorphisms in the progression of HIV-1 disease. Biochem Biophys Res Commun. 2010;396(2):348–352. 10.1016/j.bbrc.2010.04.095 20416280 PMC2897703

[20] An P, Nelson GW, Wang L, Donfield S, Goedert JJ, Phair J, et al Modulating influence on HIV/AIDS by interacting RANTES gene variants. Proc Natl Acad Sci U S A. 2002;99(15):10002–10007. 10.1073/pnas.142313799 12114533 PMC126614

[21] Winkler C, Modi W, Smith MW, Nelson GW, Wu X, Carrington M, et al Genetic restriction of AIDS pathogenesis by an SDF-1 chemokine gene variant. ALIVE Study, Hemophilia Growth and Development Study (HGDS), Multicenter AIDSCohort Study (MACS), Multicenter Hemophilia Cohort Study (MHCS), San Francisco City Cohort (SFCC). Science. 1998;279(5349):389–393. 9430590 10.1126/science.279.5349.389

[22] Brambilla A, Villa C, Rizzardi G, Veglia F, Ghezzi S, Lazzarin A, et al Shorter survival of SDF1-3'A/3'A homozygotes linked to CD4+ T cell decrease in advanced human immunodeficiency virus type 1 infection. J Infect Dis. 2000;182(1):311–315. 10.1086/315650 10882614

[23] Smith MW, Dean M, Carrington M, Winkler C, Huttley GA, Lomb DA, et al Contrasting genetic influence of CCR2 and CCR5 variants on HIV-1 infection and disease progression. Hemophilia Growth and Development Study (HGDS), Multicenter AIDS Cohort Study (MACS), Multicenter Hemophilia Cohort Study (MHCS), San Francisco City Cohort (SFCC), ALIVE Study. Science. 1997;277(5328):959–965. 9252328 10.1126/science.277.5328.959

[24] O'Brien TR, McDermott DH, Ioannidis JP, Carrington M, Murphy PM, Havlir DV, et al Effect of chemokine receptor gene polymorphisms on the response to potent antiretroviral therapy. AIDS. 2000;14(7):821–826. 10839590 10.1097/00002030-200005050-00008

[25] Fernandez S, Rosenow AA, James IR, Roberts SG, Nolan RC, French MA, et al Recovery of CD4+ T Cells in HIV patients with a stable virologic response to antiretroviral therapy is associated with polymorphisms of interleukin-6 and central major histocompatibility complex genes. J Acquir Immune Defic Syndr. 2006;41(1):1–5. 16340466 10.1097/01.qai.0000188990.57760.e3

[26] Puissant B, Roubinet F, Massip P, Sandres-Saune K, Apoil PA, Abbal M, et al Analysis of CCR5, CCR2, CX3CR1, and SDF1 polymorphisms in HIV-positive treated patients: impact on response to HAART and on peripheral T lymphocyte counts. AIDS Res Hum Retroviruses. 2006;22(2):153–162. 10.1089/aid.2006.22.153 16478397

[27] Caro-Murillo AM, Castilla J, Pérez-Hoyos S, Miró JM, Podzamczer D, Rubio R, et al [Spanish cohort of naïve HIV-infected patients (CoRIS): rationale, organization and initial results] In spanish. Enferm Infecc Microbiol Clin. 2007;25:23–31. 17261243 10.1157/13096749

[28] Sobrino-Vegas P, Gutiérrez F, Berenguer J, Labarga P, García F, Alejos-Ferreras B, et al [The Cohort of the Spanish HIV Research Network (CoRIS) and its associated biobank; organizational issues, main findings and losses to follow-up] In spanish. Enferm Infecc Microbiol Clin. 2011;29(9):645–653. 10.1016/j.eimc.2011.06.002 21820763

[29] García-Merino I, de Las Cuevas N, Jiménez JL, Gallego J, Gómez C, Prieto C, et al Spanish HIV BioBank. The Spanish HIV BioBank: a model of cooperative HIV research. Retrovirology. 2009; 6: 27 10.1186/1742-4690-6-27 19272145 PMC2667474

[30] 1000 Genomes Project Consortium, Auton A, Brooks LD, Durbin RM, Garrison EP, Kang HM, Korbel JO, Marchini JL, McCarthy S, McVean GA, Abecasis GR. A global reference for human genetic variation. Nature. 2015; 526(7571):68–74. 10.1038/nature15393 26432245 PMC4750478

[31] Solé X, Guinó E, Valls J, Iniesta R, Moreno V. SNPStats: a web tool for the analysis of association studies. Bioinformatics. 2006;22(15):1928–1929. 10.1093/bioinformatics/btl268 16720584

[32] Philpott S, Burger H, Tarwater PM, Lu M, Gange SJ, Anastos K, et al CCR2 genotype and disease progression in a treated population of HIV type 1-infected women. Clin Infect Dis. 2004;39(6):861–865. 10.1086/423386 15472820 PMC3164116

[33] Passam AM, Zafiropoulos A, Miyakis S, Zagoreos I, Stavrianeas NG, Krambovitis E, et al CCR2-64I and CXCL12 3'A alleles confer a favorable prognosis to AIDS patients undergoing HAART therapy. J Clin Virol. 2005;34(4):302–309. 10.1016/j.jcv.2004.05.021 16286054

[34] Rigato PO, Hong MA, Casseb J, Ueda M, de Castro I, Benard G, et al Better CD4+ T cell recovery in Brazilian HIV-infected individuals under HAART due to cumulative carriage of SDF-1-3'A, CCR2-V64I, CCR5-D32 and CCR5-promoter 59029A/G polymorphisms. Curr HIV Res. 2008;6(5):466–473. 18855658 10.2174/157016208785861131

[35] Lee B, Doranz BJ, Rana S, Yi Y, Mellado M, Frade JM, et al Influence of the CCR2-V64I polymorphism on human immunodeficiency virus type 1 coreceptor activity and on chemokine receptor function of CCR2b, CCR3, CCR5, and CXCR4. J Virol. 1998;72(9):7450–8. 9696841 10.1128/jvi.72.9.7450-7458.1998 PMC109977

[36] Kostrikis LG, Huang Y, Moore JP, Wolinsky SM, Zhang L, Guo Y, et al A chemokine receptor CCR2 allele delays HIV-1 disease progression and is associated with a CCR5 promoter mutation. Nat Med. 1998;4(3):350–3. 9500612 10.1038/nm0398-350

[37] Daar ES, Lynn HS, Donfield SM, Lail A, O'Brien SJ, Huang W, et al Stromal cell-derived factor-1 genotype, coreceptor tropism, and HIV type 1 disease progression. J Infect Dis. 2005;192(9):1597–1605. 10.1086/496893 16206074

[38] Oberlin E, Amara A, Bachelerie F, Bessia C, Virelizier JL, Arenzana-Seisdedos F, et al The CXC chemokine SDF-1 is the ligand for LESTR/fusin and prevents infection by T-cell-line-adapted HIV-1. Nature. 1996;382(6594):833–5. 10.1038/382833a0 8752281

[39] de Oliveira KB, Guembarovski RL, Guembarovski AM, da Silva do Amaral Herrera AC, Sobrinho WJ, Ariza CB, et al CXCL12, CXCR4 and IFNγ genes expression: implications for proinflammatory microenvironment of breast cancer. Clin Exp Med. 2013;13(3):211–9. 10.1007/s10238-012-0194-5 22699677

[40] Soriano A, Martínez C, García F, Plana M, Palou E, Lejeune M, et al Plasma stromal cell-derived factor (SDF)-1 levels, SDF1-3'A genotype, and expression of CXCR4 on T lymphocytes: their impact on resistance to human immunodeficiency virus type 1 infection and its progression. J Infect Dis. 2002;186(7):922–31. 10.1086/343741 12232832

