# Supplementary material for: Genetic variation in CCR2 and CXCL12 genes impacts on CD4 restoration in patients initiating cART with advanced immunesupression
Source: PLoS One. 2019 Mar 28;14(3):e0214421. doi: 10.1371/journal.pone.0214421 (PMC6438540; doi:10.1371/journal.pone.0214421)
Supplement: S1 Text — (PDF) [file pone.0214421.s001.pdf]

S1 Text. Centers and investigators participating in CoRIS:

Executive Committee: Santiago Moreno, Julia del Amo, David Dalmau, Maria Luisa Navarro, Maria Isabel González, Jose Luis Blanco, Federico Garcia, Rafael Rubio, Jose Antonio Iribarren, Félix Gutiérrez, Francesc Vidal, Juan Berenguer, Juan González.
Fieldwork, data management andanalysis: Paz Sobrino, Victoria Hernando, Belén Alejos, Débora Álvarez, Inma Jarrín, Nieves Sanz, Cristina Moreno.
BioBanK HIV: M Ángeles Muñoz-Fernández, Isabel García-Merino, Coral Gómez Rico, Jorge Gallego de la Fuente y Almudena García Torre.

Participating centers:

Hospital General Universitario de Alicante (Alicante): Joaquín Portilla, Esperanza Merino, Sergio Reus, Vicente Boix, Livia Giner, Carmen Gadea, Irene Portilla, Maria Pampliega, Marcos Díez, Juan Carlos Rodríguez, Jose Sánchez-Payá.

Hospital Universitario de Canarias (San Cristobal de la Laguna): Juan Luis Gómez, Jehovana Hernández, María Remedios Alemán, María del Mar Alonso, María Inmaculada Hernández, Felicitas Díaz-Flores, Dácil García, Ricardo Pelazas. , Ana López Lirola.

Hospital Universitario Central de Asturias (Oviedo): Victor Asensi, Eulalia Valle, José Antonio Cartón., Maria Eugenia Rivas Carmenado.

Hospital Universitario 12 de Octubre (Madrid): Rafael Rubio, Federico Pulido, Otilia Bisbal, Asunción Hernando, Maria Lagarde, Mariano Matarranz, Lourdes Dominguez, Laura Bermejo, Mireia Santacreu.

Hospital Universitario de Donostia (Donostia-San Sebastián): José Antonio Iribarren, Julio Arrizabalaga, María José Aramburu, Xabier Camino, Francisco Rodríguez-Arrondo, Miguel Ángel von Wichmann, Lidia Pascual Tomé, Miguel Ángel Goenaga, Mª Jesús Bustinduy, HarkaitzAzkuneGalparsoro. , Maialen Ibarguren, Maitane Umerez.

Hospital General Universitario De Elche (Elche): Félix Gutiérrez, Mar Masiá, Sergio Padilla, Andrés Navarro, Fernando Montolio, Catalina Robledano, Joan GregoriColomé, Araceli Adsuar, Rafael Pascual, Marta Fernández, Elena García., Jose Alberto García , Xavier Barber.

Hospital General Universitario Gregorio Marañón (Madrid): Juan Berenguer, Juan Carlos López Bernaldo de Quirós, Pilar Miralles, Isabel Gutiérrez, Margarita Ramírez, Belén Padilla, Paloma Gijón, Ana Carrero, Teresa Aldamiz-Echevarría, Francisco Tejerina, Francisco Jose Parras, Pascual Balsalobre, Cristina Diez.

Hospital Universitari de Tarragona Joan XXIII (Tarragona): Francesc Vidal, Joaquín Peraire, Consuelo Viladés, Sergio Veloso, Montserrat Vargas, Miguel López-Dupla, Montserrat Olona, Anna Rull, Esther Rodriguez-Gallego, Verónica Alba.

Hospital Universitario y Politécnico de La Fe (Valencia): Marta Montero Alonso, José López Aldeguer, Marino Blanes Juliá, Mariona Tasias Pitarch, Iván Castro Hernández, Eva Calabuig Muñoz, Sandra Cuéllar Tovar, Miguel SalavertLletí, Juan Fernández Navarro.

Hospital Universitario La Paz-CarlosIII-Cantoblanco: Juan González-García, F Arnalich, José Ramón Arribas, José Ignacio Bernardino, Juan Miguel Castro, Luis Escosa, Pedro Herranz, Víctor Hontañón, Silvia García-Bujalance, Milagros García López-Hortelano, Alicia González-Baeza, María Luz Martín-Carbonero, Mario Mayoral, María José Mellado, Rafael Micán, Rocío Montejano, María Luisa Montes, Victoria Moreno, Ignacio Pérez-Valero, Berta Rodés, Talia Sainz, Elena Sendagorta, Natalia C Stella y Eulalia Valencia.

Hospital UniversitariMutuaTerrassa (Terrasa): David Dalmau, Angels Jaén, Montse Sanmartí, Mireia Cairó, Javier Martinez-Lacasa, Pablo Velli, Roser Font, Mariona Xercavins, Noemí Alonso.

Hospital Universitario de La Princesa (Madrid): Ignacio de los Santos, Jesús Sanz Sanz, Ana Salas Aparicio, Cristina Sarriá Cepeda, Lucio Garcia-Fraile Fraile.

Hospital Universitario Ramón y Cajal (Madrid): Santiago Moreno, José Luis Casado, Fernando Dronda, Ana Moreno, María Jesús Pérez Elías, Cristina Gómez Ayerbe, Carolina Gutiérrez, Nadia Madrid, Santos del Campo Terrón, Paloma Martí, Uxua Ansa, Sergio Serrrano, Maria Jesús Vivancos.

Hospital General Universitario Reina Sofía (Murcia): Alfredo Cano, Enrique Bernal, Ángeles Muñoz.

Hospital Nuevo San Cecilio (Granada): Federico García, José Hernández, Alejandro Peña, Leopoldo Muñoz, Ana Belén Pérez, Marta Alvarez, Natalia Chueca, David Vinuesa, Jose Angel Fernández.

Centro Sanitario Sandoval (Madrid): Jorge Del Romero, Carmen Rodríguez, Teresa Puerta, Juan Carlos Carrió, Mar Vera, Juan Ballesteros.

Hospital Universitario Son Espases (Palma de Mallorca): Melchor Riera, Maria Peñaranda, Maria Leyes, Mª Angels Ribas, Antoni A Campins, Carmen Vidal, Francisco Fanjul, Javier Murillas, Francisco Homar.

Hospital Universitario Virgen de la Victoria (Málaga): Jesús Santos, Manuel Márquez, Isabel Viciana, Rosario Palacios, Isabel Pérez, Carmen Maria González.

Hospital Universitario Virgen del Rocío (Sevilla): Pompeyo Viciana, Nuria Espinosa, Luis Fernando López-Cortés.

Hospital Universitario de Bellvitge (Hospitalet de Llobregat): Daniel Podzamczer, Elena Ferrer, ArkaitzImaz, Juan Tiraboschi, Ana Silva, Maria Saumoy.

Hospital Costa del Sol (Marbella): Julián Olalla, Alfonso del Arco, Javier de la torre, José Luis Prada, José María García de Lomas Guerrero.

Hospital General Universitario Santa Lucía (Cartagena): Onofre Juan Martínez, Francisco Jesús Vera, Lorena Martínez, Josefina García, Begoña Alcaraz, Amaya Jimeno.

Complejo Hospitalario Universitario a Coruña (Chuac) (A Coruña): Eva Poveda, Berta Pernas, Álvaro Mena, Marta Grandal, Ángeles Castro, José D. Pedreira.

Hospital Universitario Virgen de la Arrixaca (El Palmar): Carlos Galera, Helena Albendin, Aurora Pérez, Asunción Iborra, Antonio Moreno, Maria Ángeles Campillo, Asunción Vidal.

Hospital Universitario Infanta Sofia (San Sebastian de los Reyes): Inés Suárez-García, Eduardo Malmierca, Patricia González-Ruano, Dolores Martín Rodrigo.

Complejo Hospitalario de Jaén (Jaen): Mohamed Omar Mohamed-Balghata, Maria Amparo Gómez Vidal.
