# Supplementary material for: Genetic variation in CCR2 and CXCL12 genes impacts on CD4 restoration in patients initiating cART with advanced immunesupression
Source: PLoS One. 2019 Mar 28;14(3):e0214421. doi: 10.1371/journal.pone.0214421 (PMC6438540; doi:10.1371/journal.pone.0214421)
Supplement: S1 Table — (PDF) [file pone.0214421.s002.pdf]

S1 Table. Alleles and genotypes frequencies for *CCL5* rs2280789, *CXCR2* rs1799864 and *CXCL12* rs1801157 polymorphisms in the study population compared with Iberian Populations (IBS) in Spain from 1000 genomes database (http://www.internationalgenome.org/)

SNP alleles and genotypes	All HIV infected patients (n= 412)	IBS population (n=107)	INR patients (n=134)	IR patients (n=278)	p-value[a]	p-value[b]	p-value[c]
***CCL5* rs2280789**							
**Alleles**							
A	86%	93%	85%	86%	0.07	0.08	0.08
G	14%	7%	15%	14%			
**Genotypes**							
AA	75%	85%	72%	76%	**0.04**	**0.02**	0.07
AG	23%	15%	26%	21%	0.09	0.05	0.23
GG	2%	0%	2%	3%	0.29	0.39	0.15
***CXCL12* rs1801157**							
**Alleles**							
C	78%	78%	76%	79%	0.89	0.83	0.93
T	22%	22%	24%	21%			
**Genotypes**							
CC	61%	60%	60%	62%	0.93	0.89	0.80
CT	34%	36%	32%	35%	0.80	0.60	0.94
TT	5%	4%	8%	3%	0.80	0.31	0.86
CCR2 rs1799864							
**Alleles**							
G	90%	94%	89%	91%	0.27	0.25	0.45
A	10%	6%	11%	9%			
**Genotypes**							
AA	1%	0%	0%	1%	0.66	-	0.71
GA	18%	12%	22%	16%	0.18	0.06	0.4
GG	81%	88%	78%	83%	0.12	0.06	0.29

p-values were calculated by Chi-square test; **(a)** differences between IBS population and all HIV infected patients; **(b)** differences between IBS population and INR patients; **(c)** differences between IBS population and IR patients
